# Complete Genome Sequence of the Secondary Alcohol-Utilizing Methanogen Methanospirillum hungatei Strain GP1

**DOI:** 10.1128/MRA.00708-21

**Published:** 2021-08-12

**Authors:** Leslie A. Day, Kyle C. Costa

**Affiliations:** a Department of Plant and Microbial Biology, University of Minnesota, St. Paul, Minnesota, USA; University of Arizona

## Abstract

We report the complete genome sequence of Methanospirillum hungatei strain GP1 (DSM 1101). Strain GP1 oxidizes H_2_, formate, and secondary alcohols as the substrates for methanogenesis. Members of the genus are model organisms used to study syntrophic growth with bacterial partners, but secondary alcohol metabolism remains poorly studied.

## ANNOUNCEMENT

*Methanospirillum* spp. are model organisms for studying the syntrophic growth of methanogenic archaea with partner organisms (1). They are capable of growth with H_2_ or formate as the electron donor for methanogenesis. Methanospirillum hungatei strain GP1 was isolated from a pear waste digester and, in addition to H_2_ and formate (2), can oxidize either 2-propanol or 2-butanol as the electron donor for methanogenesis (3). In methanogens, secondary alcohols are oxidized to the corresponding ketone with the concomitant reduction of coenzyme F_420_ (4, 5). This reaction is reversible and operates primarily in the direction of ketone reduction at high partial pressures of H_2_ (6). While the mechanisms of H_2_- and formate-dependent growth are well understood for hydrogenotrophic methanogens (7), the basis of alcohol utilization is underexplored. We sequenced the genome of *M. hungatei* strain GP1 to better understand how electrons from secondary alcohols feed into methanogenesis.

A liquid culture of *M. hungatei* strain GP1 was obtained from Deutsche Sammlung von Mikroorganismen und Zellkulturen (DSMZ). The material was transferred and propagated in liquid medium under an atmosphere of H_2_:CO_2_ (80:20, 140 kPa) in DSMZ medium 119 with sludge fluid omitted and 0.4% tryptone, 0.4% peptone, and 0.2% Casamino Acids added. Genomic DNA was isolated from two separate cultures using the Qiagen blood and tissue kit and submitted to the Microbial Genome Sequencing Center (https://www.migscenter.com/). One DNA extraction was used for Nanopore sequencing (ligation sequencing kit; Oxford Nanopore), and a separate DNA extraction was used for Illumina library preparation (DNA prep kit; Illumina, Inc.) and sequencing. Illumina NextSeq 2000 sequencing (151-bp paired-end reads) generated 3,715,804 reads, totaling 541,571,161 bp. The Illumina reads were trimmed using Trim Galore v. 0.6.6 (https://github.com/FelixKrueger/TrimGalore) to remove adapters and for quality control. Nanopore sequencing was performed on a MinION R9 flow cell and generated 128,156 reads, totaling 640,042,040 bp, with a read *N*_50_ value of 23,336 bp. Guppy v. 4.2.2 (Oxford Nanopore) was used for base calling. *De novo* hybrid assembly and polishing of Illumina and Nanopore reads were performed using Unicycler v. 4.9 and resulted in a single circular chromosome of 3,393,136 bp with 170× read coverage (8). The genomic G+C content is 42.2%. The assembly annotation was performed using the NCBI Prokaryotic Genome Annotation Pipeline v. 5.2 (9–11). All programs were run with default parameters.

The strain GP1 genome contains 3,139 protein coding genes and 70 RNA coding genes (genes for 55 tRNAs, 5 5S rRNAs, 4 16S rRNAs, 4 23S rRNAs, and 2 noncoding RNAs [ncRNAs]). There is a single putative coenzyme F_420_-dependent secondary alcohol dehydrogenase that shares 79% amino acid identity with the alcohol dehydrogenase of Methanoculleus thermophilus strain TCI (4). Recent data suggest that methanogens from the order *Methanomicrobiales* can oxidize F_420_H_2_ and produce formate through the action of a reversible F_420_-dependent formate dehydrogenase (12). It may be that alcohol oxidation ultimately leads to formate production before CO_2_ reduction occurs. The strain GP1 genome encodes six putative formate dehydrogenases and several hydrogenases, including F_420_-reducing hydrogenase, 5,10-methenyltetrahydromethanopterin hydrogenase (Hmd), and membrane-associated, energy-converting hydrogenases. To our knowledge, strain GP1 is the only member of the genus *Methanospirillum* known to possess Hmd.

### Data availability.

The GenBank accession number for the complete genome sequence is CP077107.1. The raw sequence reads are available in the Sequence Read Archive under the accession numbers SRX11342833 for the Nanopore reads and SRX11342832 for the Illumina paired-end reads.
